# Transcallosal Removal of a Choroid Plexus Tumor From the Lateral Ventricle in a Dog. Case Report

**DOI:** 10.3389/fvets.2020.00536

**Published:** 2020-09-29

**Authors:** László Lehner, Kálmán Czeibert, Judit Benczik, Csaba Jakab, Gábor Nagy

**Affiliations:** ^1^Fuziovet Veterinary Clinic and Hospital, Budapest, Hungary; ^2^Department of Ethology, Institute of Biology, Eötvös Loránd University, Budapest, Hungary; ^3^University of Pécs Medical School, Pécs, Hungary; ^4^Private Practitioner, Budapest, Hungary; ^5^National Institute of Clinical Neurosciences, Budapest, Hungary

**Keywords:** choroid plexus tumor, papilloma, lateral ventricle, surgery, dog

## Abstract

A 6-years-old female Staffordshire terrier was referred for periodic generalized seizures and asymmetric visual deficits. Magnetic resonance imaging revealed a 23.2 × 19.3 × 23.0 mm soft tissue mass within the right lateral ventricle and consequential dilatation of the lateral ventricles. Surgically, an interhemispheric approach was performed next to the marginal gyrus after a right parieto-parasagittal craniotomy, and a large choroid plexus tumor was transcallosally removed. After 3 days, the dog was discharged to home, and supportive treatment was continued. Histology revealed a choroid plexus papilloma, which was also confirmed by immunohistochemistry. One month after surgery, a control MRI showed that the ventricles were still dilated, but there was no sign of recurrent tumor. The dog had two additional seizures at home during the month following the intervention and one more grand mal episode was observed 4 months after the surgery. Nine months after the surgery, the dog showed no seizure activity, but her vision had not yet returned.

## Introduction

The choroid plexus (CP) is a vascular structure surrounded by ependyma. It projects into the ventricular cavities. The CP produces cerebrospinal fluid (CSF) by secretion and filtration (1, 2). Choroid plexus tumors (CPT) develop from the epithelium of the intracranial blood vessels. Based on literature, CPT are responsible for the 10% of all intracranial tumors in dogs, are usually benign tumors, and can be found in any ventricles (1–6). In humans, the most common location is the lateral ventricle in children and the fourth ventricle in adults (7–9). They account for 1–20% of brain tumors in children but only 0.5% in adults (10, 11). In dogs, CPT is observed frequently in the fourth ventricle, however, it may also appear in the third and lateral ventricles (1).

Depending on the size and location of the tumor, patients can show no clinical signs, and the neoplastic disorder is often only found in these asymptomatic cases with diagnostic imaging methods, as incidental findings (12). The clinical signs which can be observed in the patients are often the subsequent result of the hydrocephalus, which developed as a consequence of the obstruction and/or overproduction of the CSF (and the increased intracranial pressure). These clinical signs can include headaches, blindness, circling, seizures, ataxia, and behavioral changes (3, 13). In dogs, only 3% of intracranial tumors presenting with seizures are CPTs (14). Most CPTs occur in middle-aged dogs (average age 6 years, range 1–13 years) (5).

Definitive diagnosis of CPTs is achieved by histopathological assessment, although some of the masses can be identified using magnetic resonance imaging (MRI), where these tumors regularly show T2-hyperintensity and they enhance the contrast material homogeneously (5).

Ventriculomegaly occurs in 75% of the dogs with intraventricular tumors (5).

Conservative therapy or surgery is recommended following diagnosis of CPTs. Conservative therapy includes antiepileptics, steroids, pain killers, chemotherapy, and edema- and CSF production-reducing treatments (4). Based on the human recommendations when the presence of a CPT is confirmed by MRI and the patient has clinical symptoms, the primary management to be chosen is the surgical resection of the tumorous tissue, and the use of adjuvant therapy is only advised after the surgical intervention (15).

During the surgery, tumorous tissues removal is performed from lateral ventricles. The main technique is the interhemispheric-transcallosal approach in human medicine, but further interventions have been published (transcortical, neuroendoscopic approaches) where more side effects could be present (12, 16, 17). In dogs, fluorescein sodium (FS) can help determine the well-vascularized CPTs (18).

A CPP—similarly to the histological features of a normal choroid plexus—comprise bland cuboidal to columnar epithelial cells. As the CPC is usually malignant, it shows at least four of the following five characteristics: high mitotic activity, nuclear pleomorphism, necrosis, increased cellular density, and blurring of the papillary pattern (13).

According to the 2007 and 2016 World Health Organization (WHO) classification of tumors of the central nervous system, CPTs are classified as either CPPs (WHO grade I), atypical CPPs (aCPPs) (WHO grade II), or choroid plexus carcinomas (CPCs) (WHO grade III) (4, 5, 9, 10, 13, 19, 20). Using the WHO classification system to categorize human CPTs, it could be verified that the grade of a tumor can be considered as the most important postsurgical prognostic factor (5).

Following the human recommendations, we decided to perform surgical removal of a CPT, and as the inter-hemispheric approach was known to have fewer postoperative side effects (compared to the transcortical surgeries), we chose this way to expose the ventricular site. To the best of the authors' knowledge there are no reports on transcallosal lateral ventricular tumor removal with 9-months follow-up in the veterinary literature.

## Case Description

A 6-years-old female Staffordshire terrier was referred for periodic generalized seizures and asymmetric visual deficit. Due to her neurologic signs, the local vet recommended an MRI examination, which was performed using a 3 Tesla MRI scanner (Siemens Magnetom Prisma 3T with a Siemens Head/Neck 20 3T TIM coil, Erlangen, Germany).

Before scanning, the dog was sedated using a 22 G intravenous catheter (Vygonüle V Luer-Lock; Laboratories Pharmaceutiques Vygon, France) that was placed in the lateral saphenous vein of the right hind limb. The dog was premedicated with medetomidine-hidroklorid (0.5 mL Narco Start 1 mg/mL A.U.V., Le Vet B.V., The Netherlands). Following premedication, anesthesia was induced with 2 mg/bwkg propofol intravenously (Propofol 1% MCT/LCT, 5.5 mg/bwkg, Fresenius-Kabi). The following sequences were made: T2-weighted sagittal view of the head; FLAIR transversal; and pre- and post-contrast T1-weighted MPRAGE.

**Figure 1:**
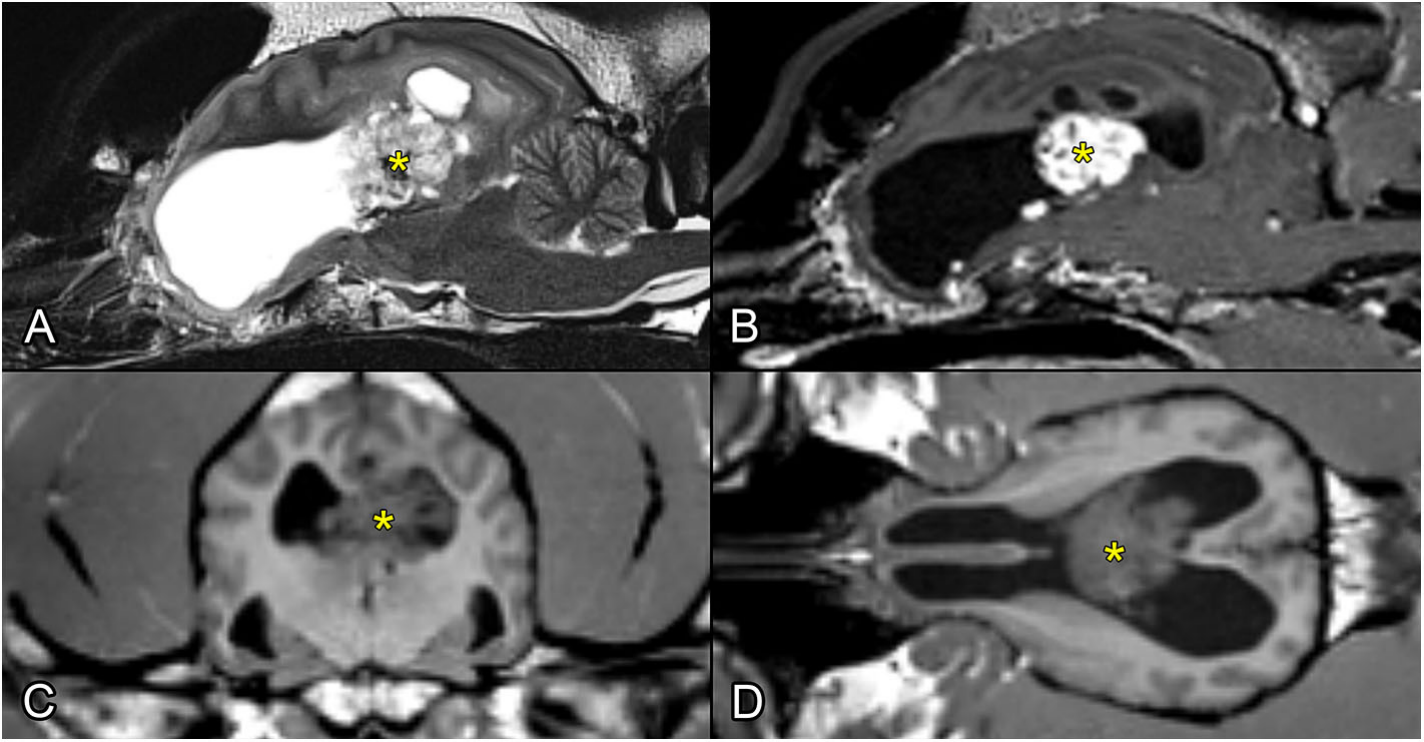
Preoperative MR images showing the intraventricular tumor (with yellow asterisk). **(A)** T2-weighted image, sagittal plane. **(B)** T1-weighted postcontrast image, sagittal plane. **(C)** T1-weighted image, transverse plane. **(D)** T1-weighted image, dorsal plane.

The MRI revealed that the lateral ventricles were moderately enlarged, and dorsal to the lateral ventricles at the midline, there were three fluid-filled cystic structures. The most caudal was 11.5 × 7.5 mm, the cranial was 4.3 mm, and the middle was 8.5 × 2.4 mm in diameter. The plexus choroideus almost completely occupied the middle region of the right lateral ventricle and invaded the left lateral ventricle. The plexus choroideus had irregular borders and a “cauliflower-like” appearance. The proliferating choroid plexus on T1- and T2-weighted images was isointense with the cerebral gray matter, while on FLAIR images, it was slightly hyperintense. On all sequences it contained well-circumscribed areas of low signal intensity. It was intensely enhanced on the post-contrast T1-weighted images. On the FLAIR images, there was a 5 × 6 mm area of increased signal intensity medial to the pars centralis of the right lateral ventricle (caudal to the largest cystic structure). The optic nerves with their surroundings and the cerebellum were normal (Figure 1). Presumed radiological differential diagnosis included the following diseases: choroid plexus papilloma, choroid plexus carcinoma, atypical choroid plexus papilloma, ependymoma, astrocytoma (subependymal giant cell or pilocytic).

After the MRI, the dog was referred for neurologic examination and surgery. Neurologic examination revealed an absent menace response and direct pupillary reflex on the right. Other cranial reflexes were normal, mental state, behavior, body posture and gait did not show any abnormalities. Proprioception and hopping tests were unremarkable. All spinal reflexes were normal. Blood and urine tests revealed no abnormalities. Abdominal and heart ultrasound findings were normal. A survey chest radiograph showed no sign of metastasis. According to the neurological examination and history the neurological localization was left sided occipital lobe, multifocal lesions in the brain. Following differential diagnosis were presumed. Vascular (ischemic) event, inflammation (MUO/MUE), anomaly (hydrocephalus), idiopathic epilepsy, neoplasia with or without altered fluid circulation.

During the surgery, the dog was positioned in sternal recumbency, and the head was elevated to ensure blood outflow. Fentanyl (5 μg/bwkg, Richter Gedeon), dormicum (0.05 mg/bwkg, EGIS), and ketamine (CP Ketamin 10% injection AUV, Medicus Partner) injections were used for premedication. The induction drug was propofol (Propofol 1% MCT/LCT, 5.5 mg/bwkg, Fresenius-Kabi), and anesthesia was maintained with a mixture of isoflurane and oxygen gas (Isoflutek 1,000 mg/g, 1.5 v/v %, Laboratorios Karizoo). During the surgery, fentanyl-ketamine (Fentanyl: 5 μg/bwkg, Richter Gedeon, Ketamin: CP Ketamin 10% injection AUV, Medicus Partner) infusion was maintained with an infusion pump (1 mL fentanyl + 0.06 mL ketamine/100 mL infusion with a rate of 100 mL/10 bwkg/h). The hair on the head was shaved off, and the head was prepared by the aseptic method. Median skin incision was performed from the protuberantia occipitalis externa to the point of Bregma. The middle and caudal auricular muscle groups midline origine have been transsected, so does the temporal muscle, which was removed from the parieto-temporal area of the skull, starting the incision next to the external sagittal crest. A right parieto-parasagittal craniotomy was performed with an oscillating saw (Stryker TPX, Stryker Inc, Kalamazzo, USA) device. The craniotomy did not pass through the midline, we kept 14 mm distance paralel to the external sagittal crest. We made a quadrangular craniotomy with a size of 32 × 24 mm on the parietal bone. The separated bone fragment was placed immediately into saline solution.

**Figure 2:**
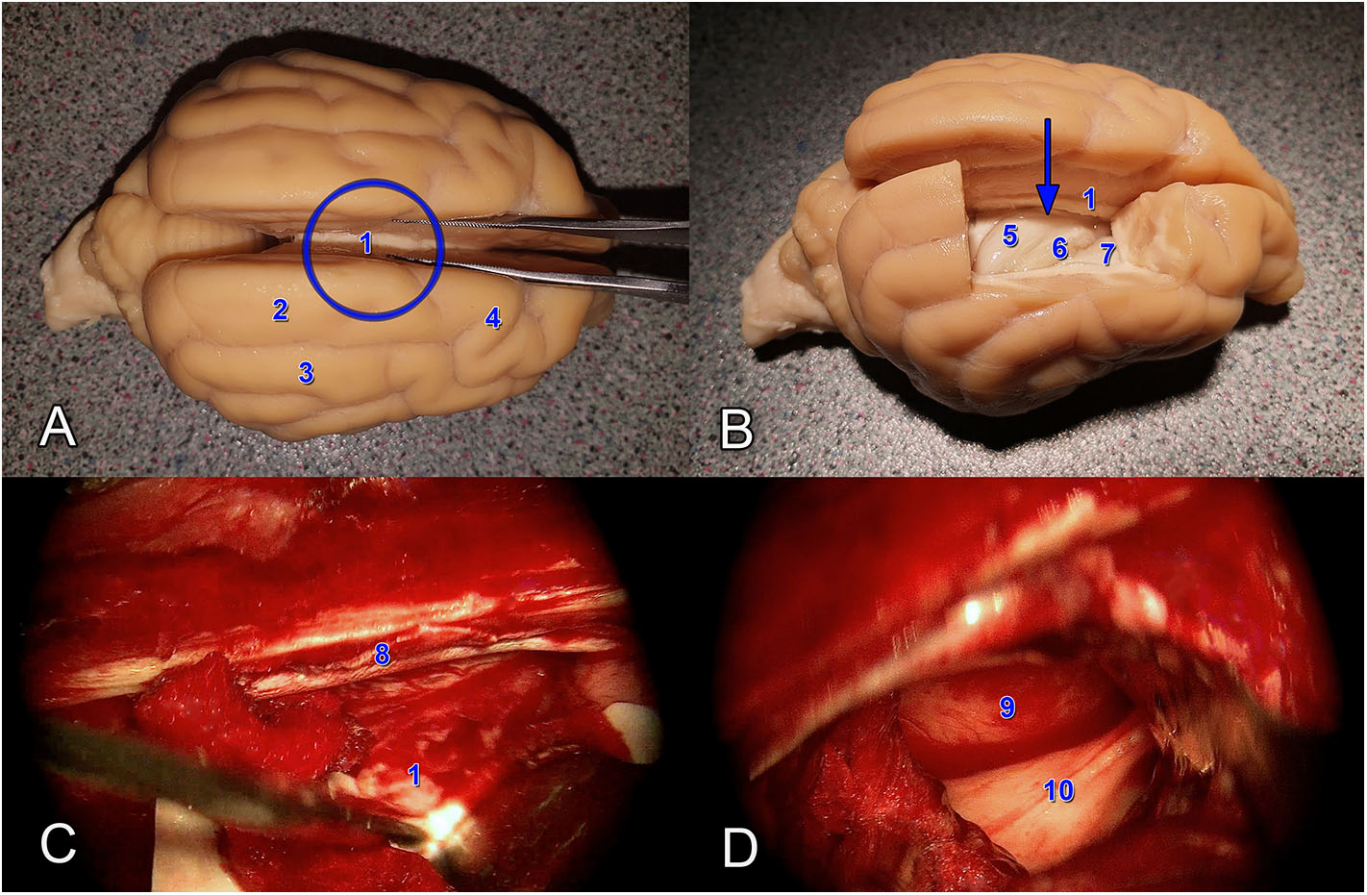
Anatomical demonstration **(A,B)** and intraoperative images **(C,D)** showing the transcallosal approach. The blue circle shows the site, whilst the blue arrow shows the direction of the approach. (1) Corpus callosum. (2) Gyrus marginalis. (3) Gyrus ectomarginalis. (4) Gyrus postcruciatus. (5) Hippocampus. (6) Plexus chorioideus. (7) Nucleus caudatus dexter. (8) Falx cerebri. (9) Nucleus caudatus sinister. (10) Corpus fornicis.

After the craniotomy, a durotomy was made using an No. 11 blade to make a 4-sided flap. The opening of the dura mater revealed a part from the right marginal to ectomarginal gyrus. Fluid was drained off by 21G intravenous catheter (VasoFix Branüle, BBraun, Germany) from the trigonal region in order to relax the brain for dissection. With a careful approach between the falx cerebri and marginal gyrus (thus the dorsal sagittal sinus was not exposed and remained protected), then deeper between the left and right cingulate gyri, the corpus callosum was reached. The marginal and ectomarginal gyrus were bluntly dissected, and the gyri (including the cingulate gyrus) was held away with brain spatulas to maintain the field of view (Figure 2). The corpus callosum was then transected with the spatula and forceps at its midline in the midsagittal plane.

After controlling subsequent bleeding with gelatine sponge (Spongostan Standard 7 × 5 × 1 cm, Ethicon Inc., New Jersey, USA) and bipolar electrocauter (Yasargil Coagulation Forceps, Aesculap Inc., Center Walley, USA), the surface of the tissue mass could be seen. The entire intracranial procedure was supported by an operation microscope (Topcon OMS-90, USA). The tumor was removed with 8- and 12-Ch Fergusson suction cannulas (Aesculap, USA) in multiple fragments until the point when there was no bleeding or visible residual tumor tissue.

The ventricle was filled with body heat sterile Salsol solution (Salsol infusion, TEVA, Hungary). The dura mater flaps were sutured with a 3/0 polyfilament suture (Surgicryl, SMI, St. Vith, Belgium), and the craniotomy fragment was attached to the neighboring bones by drilling one hole on each side of the fragment and the adjacent bones, and anchoring them with a 2/0 polyfilament suture (Surgicryl, SMI, St. Vith, Belgium). The temporal muscle and the superficial auricular muscles were sutured along the midline cut line to their other sided counterparts. The skin was closed in two layers with subcutaneous and cutaneous sutures.

After the surgery, methylprednisolone (Solu-MEDROL injection, 2 mg/bwkg, Pfizer, USA), cefazolin (Cefazolin-Sandoz, 30 mg/bwkg, Sandoz GmbH, Germany), morphine hydrochloride (Morphinum Hydrochloricum, TEVA 20 mg/mL injection, 2–4 mg/bwkg, TEVA, Israel), and mannitol (Mannisol TEVA infusion, 1 g/bwkg, TEVA, Israel) were administered. Strict observation was performed for 24 h, during which period body temperature, heart rate, respiratory rate, proprioception, cranial reflexes, and mental state were registered. During this observation period, there was an interval when the aforementioned parameters had a higher variance: arrhythmia, decreased heart and breath rate, salivation, absent pupillary light reflex, and absent palpebral reflex. As intervention for this emergency situation, epinephrine (Tonogen 1 mg/mL injection, 0.02 mg/bwkg, Richter Gedeon, Hungary) was administered, resulting in the majority of symptoms resolving except for the excited behavior, absent menace response, and twitching of the right upper lip. The excited behavior improved with midazolam (Dormicum 5 mg/mL injection, 0.5–1 mg/bwkg, EGIS, Hungary).

Neurologic examination on the following day showed an absent menace response on both sides and mild sign of weakness and the dog was dysphoric. The previously absent right sided direct PLR became normal. Mild ventrolateral strabismus was seen on the right side. Further cranial and spinal reflexes did not show any abnormalities. Mild confusion was observed. Behavior, body posture and gait were normal. Proprioception and hopping tests were unremarkable. Bilateral occipital lobe lesion was presumed according to the neurological examination. The dog was discharged after 3 days. During this period, the dog was dysphoric, but this improved with midazolam (Dormicum 5 mg/mL injection, 0.5–1 mg/bwkg, EGIS, Hungary).

At-home treatment was continued with prednisolone for 1 month (Prenisolon-Richter 5 mg tablet, 1 mg/bwkg BID, Richter Gedeon, Hungary), amoxicillin-clavulanic acid for 1 week (Augmentin 500 mg/125 mg tablet, 20 mg/bwkg BID, GlaxoSmithKline, UK), tramadol for 1 week (Tramadol LA tablet, 2–4 mg/bwkg BID, Aluid Pharma, Germany), and levetiracetam continuously (Levetiracetam TEVA 500 mg tablet, 10 mg/bwkg BID, TEVA, Israel).

**Figure 3:**
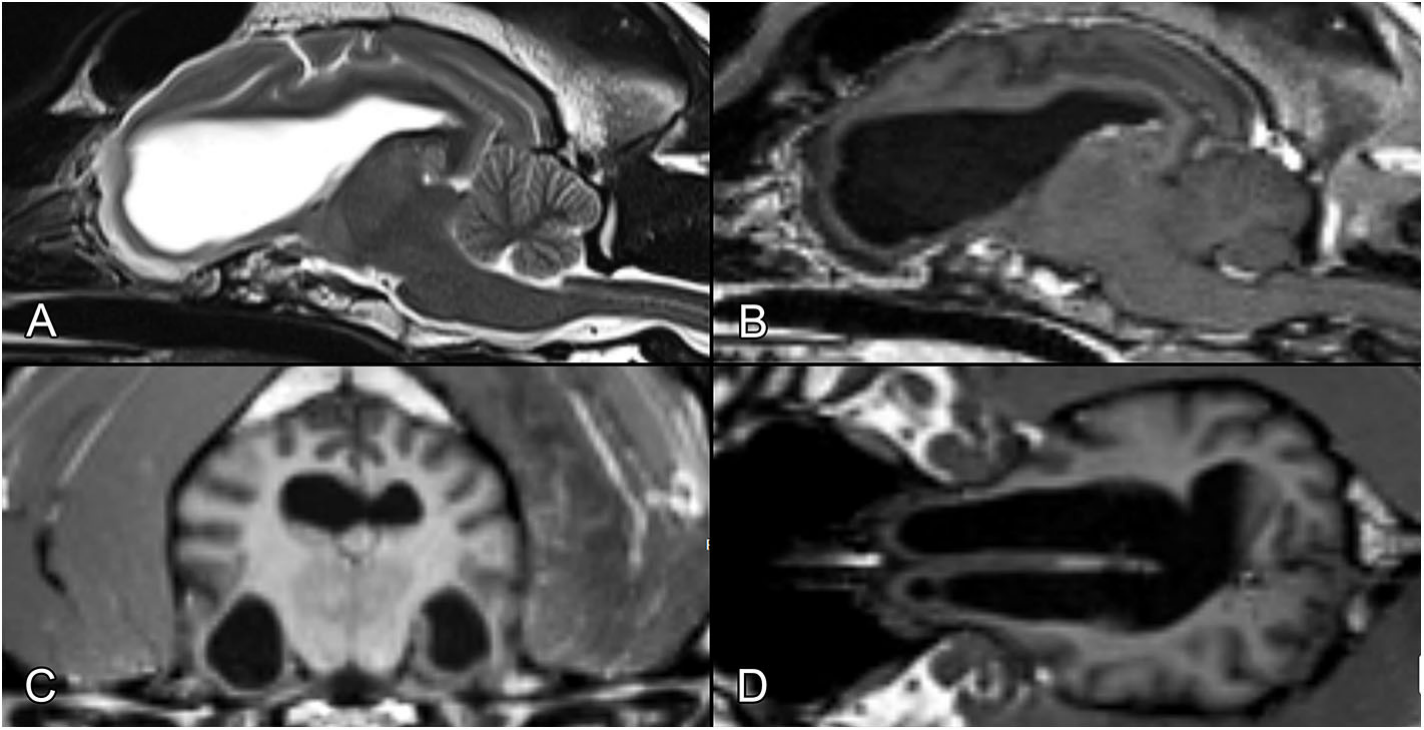
Postoperative MR images after the removal of the tumor, at the same levels as in Figure 1. **(A)** T2-weighted image, sagittal plane. **(B)** T1-weighted postcontrast image, sagittal plane. **(C)** T1-weighted image, transverse plane. **(D)** T1-weighted image, dorsal plane.

Within 1 month of intervention, the dog had two tonic-clonic seizures lasting 5 min each. One month after surgery, control MR examination was performed with the aforementioned machine using the following sequences: T2-weighted sagittal, transversal, and coronal imaging of the head; FLAIR, SWI, and mIP transversal imaging of the brain; and pre- and post-contrast T1-weighted imaging of the cranium in the sagittal, transversal, and coronal planes. The lateral ventricles were moderately enlarged, as in the preoperative examination, but the three fluid-filled cystic structures dorsal to the lateral ventricles at the midline were no longer present. The proliferating CPT seen earlier was gone. The intravenously administered contrast agent appeared in the area of the surgery and vasculature on the right area of parieto-occipital region. On the T1- and T2-weighted sequences, ventral to the pars centralis of the lateral ventricles, the well-circumscribed hyperintense areas with a hypointense rim on the T2-weighted sequences were most likely late subacute hemorrhages. The cerebellum was normal (Figure 3).

**Figure 4:**
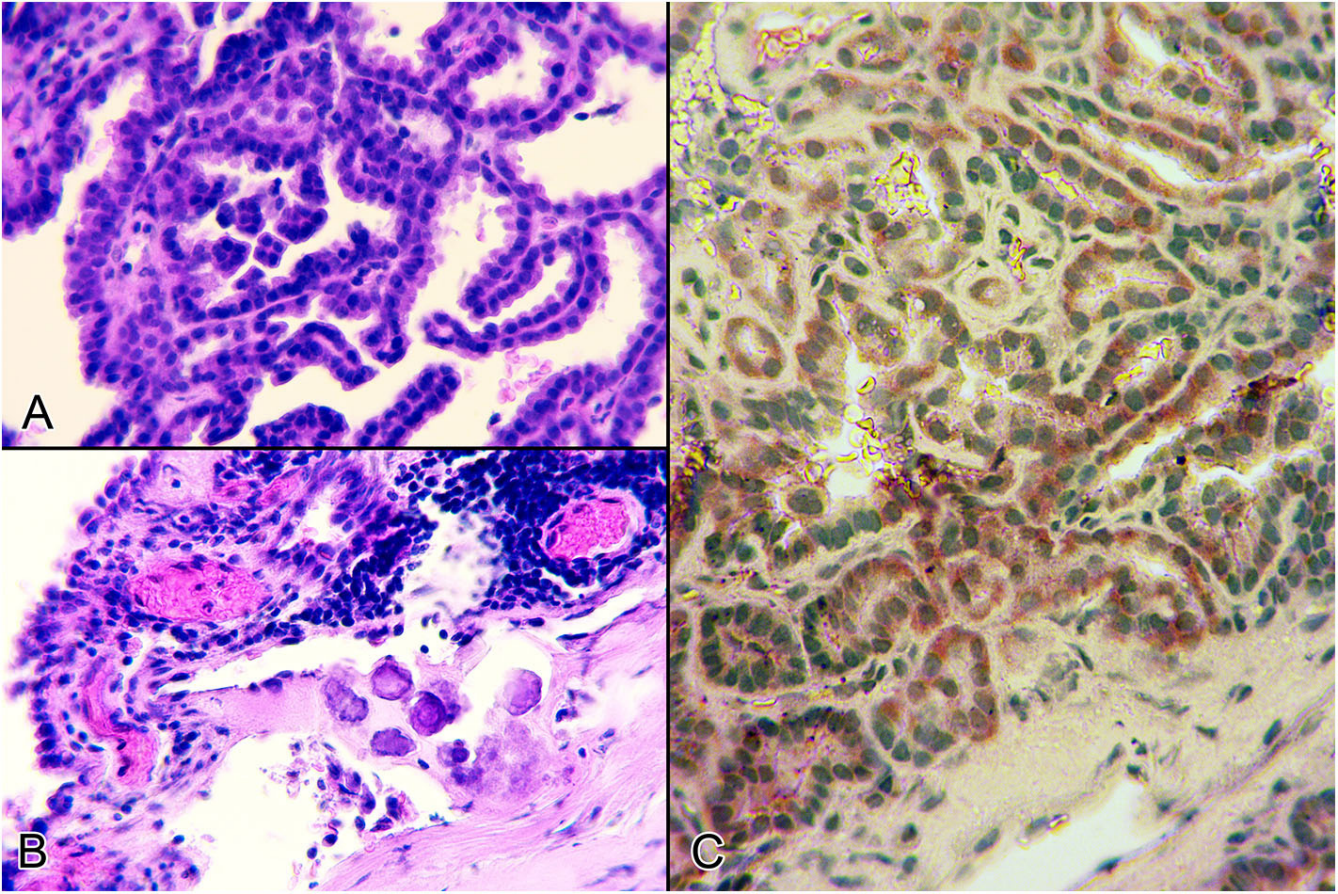
Histological sections from the intraventricular tumor. **(A)** Microscopic picture about the tumor which consisted of papillae lined by a single layer of cuboidal or columnar epithelium (Haematoxylin and eosin stain, 400x). **(B)** Lymphocytic reactivation, multifocal microcalcification and vasodilatation int he stroma of the tumor (Haematoxylin and eosin stain, 400x). **(C)** Diffuse, intense, pancytokeratin-positivity in the benign neoplastic epithelial cells of the choroid plexus papilloma (IHC, 400x).

Three months after the surgery, the menace response was still absent, but no more epileptic events were observed, and the dog's general state was stable. Levetiracetam dosage was tapered to 5 mg/bwkg BID and prednisolone was tapered to 0.5 mg/bwkg and administration of steroid was stopped 1 month after the surgery. Four months after the intervention two grand mal episodes were observed. Due to reappeared epilepsy activity, Levetiracetam dosage was increased to 20 mg/bwkg. During the 9 months postoperative follow-up, no more epilepsy events were detected and her vision had not yet returned. Histopathologic examination showed fronds of the differentiated fibrovascular tissue covered by one layer of uniform cuboidal and columnar epithelial cells (Figure 4A). No nuclear, cellular atypia, intratumoral necrosis, apoptosis, or mitosis were seen. The tumor tissue did not infiltrate, but there was compressed adjacent, peritumoral neural tissue, where we could detect mild edema and hemorrhage. Multifocal microcalcification and xanthoma production, furthermore focal lymphocytic reactivation, were detected in the stroma of the tumor (Figure 4B). The epithelial cells showed intense, diffuse, homogeneous cytoplasmatic-pancytokeratin-positivity (Figure 4C). The Ki-67-labeling-index was 1–2%. A benign CPP was ultimately diagnosed.

## Discussion

To the best of the authors' knowledge, this is the first case report about a successful removal of a CPT from the lateral ventricle in a dog. Different surgical techniques for CPT removal have been described in the human literature. Historically, transcortical approaches have been favored over interhemispheric routes. More recently however, interhemispheric transcallosal approaches have gained popularity because, in this way, no cortical brain tissue should be damaged to provide direct access to the ventricular system (12, 16, 17). In humans the treatment of choice for CPTs is surgical removal. Complete resection is curative and can well be achieved, therefore palliative shunting to treat only raised intracranial pressure caused by hydrocephalus is not an option (15). In our case, a transcallosal technique was used.

The approach was through the corpus callosum, and it affected the middle third of the marginal gyrus. This region is adjacent to the visual and somatosensory cortices, comprising the secondary, association fields of these regions (21, 22). In the surrounding white matter, one can find the superior longitudinal fasciculus (connecting the occipital and frontal poles), the cingulum (running deep to the cingulate gyrus), and the subcallosal fasciculus (similar to the cingulum, but it also gives fibers to the caudate nucleus) (21, 22). Damage to the above-mentioned structures may result in altered sensation, psychic symptoms (due to the limbic system's involvement), or unilateral central visual impairment. The most important intraventricular structures that may be damaged during the surgery are the hippocampi. Unilateral damage is tolerable, but bilateral hippocampal loss leads to impair spatial memory (23, 24).

One interesting feature was regarding the change in the PLR. While before surgery the right eye did not respond to the direct PLR, but did respond to the indirect PLR, postoperatively this condition normalized, and both direct and indirect PLR were present. The left eye was unaffected throughout. As the right direct PLR has returned, it means that no irreversible change has happened in the optic pathway. Thus, due to the normal indirect PLR on both sides the optic nerves and the optic tracts had to be unaffected (and they are running on the basal and lateral aspect of the brain, so the tumor could not compress them directly either). The oculomotor nerve and its parasympathetic nucleus had to be also intact to convey the impulse arriving from both sides. Subsequently, a possible explanation for that phenomenon could be that the left sided pretectal nucleus was under an influence which affected its signal transmission to the right sided parasympathetic oculomotor nucleus. As the vast majority of the fibers to the Edinger-Westphal nucleus arrive from the contralateral pretectal nucleus, but a minor portion is conveyed through the ipsilateral pretectal area, it could explain why the direct PLR was absent (or severely reduced) initially, and normalized after the surgery. In addition, the neoplastic tissue is also located on the dorsal aspect of the brainstem, where the pretectal area can be found.

Prior the surgery on the right sided absent menace response was occurred by chronic asymmetrical dilated ventricles which may have caused alteration of left sided occipital lobe and optic radiation tract. These alterations have been observed in human medicine due to severe hydrocephalus-like main symptoms (13). Postoperative left sided menace response was absent due to the iatrogenic damage of optic radiation. These were also observed on the right parieto-occipital lobes on the postoperative MRI, and the subsequent visual deficit could be the consequence of the damage of the central visual pathway. On the right side the previously absent menace response remained because of the chronic dilatation effect of the hydrocephalus.

Perioperative mortality due to uncontrollable hemorrhage during resection ranges from 0 to 30% (8, 12). As in humans, the main intraoperative complication in our case was bleeding from the CPT (8). It was controlled with thermocoagulation and specific anticoagulation materials (8, 19). Observation is most critical in the 24 h following surgery, especially in cases of intraventricular tumor removal (12). The most prominent postoperative complications are subdural or epidural hemorrhage, intracranial pressure elevation, edema, or clot formation (12). Our assumption is that a clot caused a CSF outflow obstruction and secondary intracranial pressure elevation in the dog, resulting in her temporarily feeling unwell. In the 1st month, seizure activity may be the result of the change in the CSF circulation and the remodeling of vascular structures. Cortical incisions have more frequently lead to epileptic seizures than transcallosal incisions in human medicine (25–27). Levetiracetam was administered after the surgery to reduce the secondary effects of vascular damage and CSF circulation disturbance (28). We chose this antiepileptic because it can prevent the effects of scar formation caused by damaged nervous tissue, decreasing the chance of epilepsy (28).

According to the human data, total resection is the most effective treatment for CPTs, but it is challenging due to the major surgical risk caused by these tumors' high vascularity (11, 19, 29). The efficacy of the surgical removal of a CPT can be considered as the single most determinant factor for long-term survival (19). In one human study, tumor recurrence was observed in 25% of patients (19). Information about surgical risks and follow-up have not been published in veterinary literature. In one study, a 4-years-old female French bulldog was treated conservatively with prednisolone, a 10% glycerin, 5% fructose mixture, acetazolamide, and hydroxyurea for 1 month (4). At first the dog improved, but 15 months after presentation, it developed a coma and died despite intensive treatment.

Ventricular enlargement that develops secondary to a CPT will not change following intervention because dilation due to disturbed fluid outflow is chronic (19). Other signs of hydrocephalus and ICP elevation on MRI are periventricular edema in FLAIR sequences, dilatation of the olfactory recess, elevation of the corpus callosum and dorsoventral flattening of the interthalamic region, thinning of the cortical sulci and subarachnoid place, ventricle/brain index >0.6 on dorsal plane images (30). In our case, aforementioned signs of ICP elevation were not detected due to the chronic state of hydrocephalus. In certain cases, when chronic ventricular dilation causes neurologic signs, permanent ventriculoperitoneal shunt (VPS) placement is recommended following tumor removal (25).

In summary, the presented transcallosal approach in dogs appears to be a safe alternative to the transcortical approach. Its advantage is the better visualization and therefore safer radical removal of those intraventricular tumors that are located close to the midline, either in the lateral ventricles pars centralis or in the third ventricle. If asymmetric symptoms (e.g., visual deficit) are seen during preoperative examination, a surgical approach is recommended to be made on the same side where the cerebral lesion is presumed, in order to avoid the possible occurrence of bilateral cortical lesion and subsequent visual deficit. Our patient was monitored through a 9-months-long follow-up period, and at the time of the submission of the manuscript her state is stable and seizure-free. Further cases are needed to build a relevant conclusion from transcallosal technique.

## Data Availability Statement

All datasets generated for this study are included in the article/supplementary material.

## Ethics Statement

Ethical review and approval was not required for the animal study because this study was carried out in accordance with regulations of the Hungarian Animal Health Care and guidelines of the veterinary science. As we report a case study of veterinary hospital we have the consent of both the owner and veterinarian that this dog (underwent the listed examinations and surgical intervention) was treated not for experimental but for medical reason. Due to the latter there was no need for approval of an ethical committee. Written informed consent was obtained from the owners for the participation of their animals in this study.

## Author Contributions

LL, GN, and KC: concept, original draft, collecting data, editing, and reviewing draft. JB and CJ: collecting data, editing, and reviewing draft. All authors contributed to the article and approved the submitted version.

## Conflict of Interest

The authors declare that the research was conducted in the absence of any commercial or financial relationships that could be construed as a potential conflict of interest.

## References

[1] Pinto ACBCF Villamizar LA Ghirelli CO Silva TRC Baroni CO Banon GPR Choroid plexus papilloma in a Rottweiler: computed tomographic, gross morfological and histological features. Arquivo Brasileiro de Medicina Veterinária e Zootecnia. (2013) 65:763–7. 10.1590/S0102-09352013000300023

[2] de Castro FD Reis F Guerra JGG. Intraventricular mass lesions at magnetic resonance imaging: iconographic essay - part 1. Radiol Bras. (2014) 47:176–81. 10.1590/0100-3984.2013.1696 25741075 PMC4337139

[3] Oura TJ Early PJ Jennings SH Lewis MJ Tobias JR Thrall DE Canine choroid plexus tumor with intracranial dissemination presenting as multiple cystic lesions. Case Rep Vet Med. (2013) 2013:759054 10.1155/2013/759054

[4] Itoh T Uchida K Nishi A Shii H Nagayoshi T Sakamoto H. Choroid plexus papilloma in a dog surviving for 15 months after diagnosis with symptomatic therapy. J Vet Med Sci. (2016) 78:167–9. 10.1292/jvms.15-0330 26321300 PMC4751140

[5] Westworth DR Dickinson PJ Vernau W Johnson EG Bollen AW Kass PH. Choroid plexus tumors in 56 dogs (1985-2007). J Vet Intern Med. (2008) 22:1157–65. 10.1111/j.1939-1676.2008.0170.x 18691364

[6] Dalton MF Stilwell JM Krimer PM Miller AD Rissi DR. Clinicopathologic features, diagnosis, and characterization of the immune cell population in canine choroid Plexus tumors. Front Vet Sci. (2019) 6:224. 10.3389/fvets.2019.00224 31380398 PMC6646530

[7] Waldron JS Tihan T. Epidemiology and pathology of intraventricular tumors. Neurosurg Clin N Am. (2003) 14:469–82. 10.1016/S1042-3680(03)00060-3 15024796

[8] Asmaro K Pawloski J Skoch J. Giant choroid plexus papilloma resection utilizing a transcollation system. Oper Neurosurg. (2019) 18:47–51. 10.1093/ons/opz096 31065711

[9] Dash C Moorthy S Garg K Singh PK Kumar A Gurjar H. Management of choroid plexus tumors in infants and young children up to 4 years of age: an institutional experience. World Neurosurg. (2019) 121:e237–45. 10.1016/j.wneu.2018.09.089 30261376

[10] Bahar M Hashem H Tekautz T Worley S Tang A de Blank P. Choroid plexus tumors in adult and pediatric populations: the Cleveland Clinic and University Hospitals experience. J Neurooncol. (2017) 132:427–32. 10.1007/s11060-017-2384-1 28290001

[11] Lin H Leng X Qin C Du Y Wang W Qiu S. Choroid plexus tumours on MRI: similarities and distinctions in different grades. Cancer Imaging. (2019) 19:17. 10.1186/s40644-019-0200-1 30894223 PMC6427869

[12] Anderson RC Ghatan S Feldstein NA. Surgical approaches to tumors of the lateral ventricle. Neurosurg Clin N Am. (2003) 14:509–25. 10.1016/S1042-3680(03)00054-8 15024798

[13] Smith AB Smirniotopoulos JG Horkanyne-Szakaly I. From the radiologic pathology archives: intraventricular neoplasms: radiologic-pathologic correlation. Radiographics. (2013) 33:21–43. 10.1148/rg.331125192 23322825

[14] Schwartz M Lamb CR Brodbelt DC Volk HA. Canine intracranial neoplasia: clinical risk factors for development of epileptic seizures. J Small Anim Pract. (2011) 52:632–7. 10.1111/j.1748-5827.2011.01131.x 21954970

[15] McEvoy AW Harding BN Phipps KP Ellison DW Elsmore AJ Thompson D. Management of choroid plexus tumours in children: 20 years experience at a single neurosurgical centre. Pediatr Neurosurgr. (2000) 32:192–9. 10.1159/000028933 10940770

[16] Ellenbogen RG. Transcortical surgery for lateral ventricular tumors. Neurosurg Focus. (2001) 10:E2. 10.3171/foc.2001.10.6.3 16724820

[17] Cikla U Swanson KI Tumturk A Keser N Uluc K Cohen-Gadol A. Microsurgical resection of tumors of the lateral and third ventricles: operative corridors for difficult-to-reach lesions. J Neurooncol. (2016) 130:331–40. 10.1007/s11060-016-2126-9 27235145 PMC5090015

[18] Nakano Y Nakata K Shibata S Heishima Y Nishida H Sakai H. Fluorescein sodium-guided resection of intracranial lesions in 22 dogs. Vet Surg. (2018) 47:302–9. 10.1111/vsu.12763 29247539

[19] Hosmann A Hinker F Dorfer C Slavc I Haberler C Dieckmann K. Management of choroid plexus tumors-an institutional experience. Acta Neurochir. (2019) 161:745–54. 10.1007/s00701-019-03832-5 30783805 PMC6431303

[20] Cantile C Campani D Menicagli M Arispici M. Pathological and immunohistochemical studies of choroid plexus carcinoma of the dog. J Comp Pathol. (2002) 126:183–93. 10.1053/jcpa.2001.0544 11945007

[21] Evans EH de Lahunta A Miller's Anatomy of the Dog. 4th ed. Elsevier (2012). Available online at: https://www.amazon.de/Millers-Anatomy-Dog-Howard-Evans/dp/1437708129?ie=UTF8&tag=googdemozdesk-21&hvadid=355868911830&hvpos=&hvnetw=g&hvrand=13160362630918366984&hvpone=&hvptwo=&hvqmt=b&hvdev=c&hvdvcmdl=&hvlocint=&hvlocphy=9063088&hvtargid=dsa-19959388920&ref=pd_sl_6z0p6oc4rh_e&language=en_GB (accessed March 13, 2020).

[22] De Lahunta A Glass E Kent M Veterinary Neuroanatomy and Clinical Neurology. 4th ed. Elsevier (2014) Available online at: https://www.elsevier.com/books/veterinary-neuroanatomy-and-clinical-neurology/de-lahunta/978-1-4557-4856-3 (accessed March 13, 2020).

[23] Kowalska DM. Effects of hippocampal lesions on spatial delayed responses in dog. Hippocampus. (1995) 5:363–70. 10.1002/hipo.450050409 8589799

[24] Kowalska DM. Cognitive functions of the temporal lobe in the dog: a review. Prog Neuropsychopharmacol Biol Psychiatry. (2000) 24:855–80. 10.1016/S0278-5846(00)00110-X 11191717

[25] Dănăilă L. Primary tumors of the lateral ventricles of the brain. Chirurgia. (2013) 108:616–30. 24157104

[26] Ratha V Kumar VRR. Transventricular migration of choroid plexus carcinoma causing an intraoperative conundrum: a case report with a review of the literature. Pediatr Neurosurg. (2019) 54:341–6. 10.1159/000500300 31536979

[27] Elwatidy SM Albakr AA Al Towim AA Malik SH. Tumors of the lateral and third ventricle: surgical management and outcome analysis in 42 cases. Neurosciences. (2017) 22:274–81. 10.17712/nsj.2017.4.20170149 29057852 PMC5946376

[28] Kennewell P Comprehensive Medicinal Chemistry II, Volume 1: GLOBAL PERSPECTIVE. 1 Edn. Amsterdam: Elsevier Science (2006).

[29] Cao L-R Chen J Zhang R-P Hu X-L Fang Y-L Cai C-Q. Choroid plexus papilloma of bilateral lateral ventricle in an infant conceived by *in vitro* fertilization. Pediatr Neurosurg. (2018) 53:401–6. 10.1159/000491639 30391955

[30] Mai W Diagnostic MRI in Dogs and Cats. CRC Press (2018). Available online at: https://www.routledge.com/Diagnostic-MRI-in-Dogs-and-Cats-1st-Edition/Mai/p/book/9781498737708 (accessed June 22, 2020).

